# A Mathematical Framework for Determining the Effect of Rotational Errors on Single-Isocenter Multi-target Stereotactic Radiosurgery

**DOI:** 10.7759/cureus.111848

**Published:** 2026-06-30

**Authors:** Nidhir S Narayanan, Andrew W Suen, Huong T Pham, Sreeram Narayanan

**Affiliations:** 1 Medicine, The Overlake School, Redmond, USA; 2 Radiation Oncology, Virginia Mason Medical Center, Seattle, USA

**Keywords:** linac-based radiosurgery, rotational corrections, single-isocenter multiple-target srs, volumetric-modulated arc-based radiosurgery

## Abstract

Introduction

Single-isocenter multi-target (SIMT) radiosurgery enhances efficiency by treating multiple lesions with a single plan. However, this technique is highly susceptible to patient positioning errors, particularly rotational deviations. Even minor rotations can lead to significant reductions in target dose coverage, with the degree of degradation being influenced by target size, the magnitude of rotation, and the target's distance from the treatment isocenter. While previous studies have investigated these effects through simulations, retrospective analyses, or statistical models, a robust mathematical framework for predicting these interactions is lacking.

Purpose

This study aims to develop and apply mathematical models to quantify the impact of rotational errors on target coverage in SIMT radiosurgery. The primary objective is to elucidate the interplay between target size, distance from the isocenter, and the magnitude of rotational error, with a view to informing individualized planning target volume (PTV) margin selection for optimized target coverage and minimized normal tissue irradiation.

Methods

We developed mathematical models to simulate the dosimetric consequences of rotational errors under the assumption of a dose distribution encompassing the target perfectly, in the absence of setup errors. The target and radiation fields were modeled as three-dimensional spheres, and equations were derived to calculate the volume of intersection between the rotated target and the prescribed radiation field. The percentage of target volume maintained within the prescription dose sphere was then calculated. Graphical representations of Gross Tumor Volume (GTV) coverage as a function of rotational error (ranging from 0° to 3°), target diameter (0.5 cm to 3.0 cm), PTV margins (0 mm, 0.5 mm, 1.0 mm), and distance from the isocenter (1.5 cm to 7.5 cm) were generated. The methodology was applied to clinical cases to demonstrate its utility in deriving customized margins.

Results

The mathematical analysis demonstrated that small targets located further from the isocenter are most vulnerable to coverage degradation due to rotational errors, especially with minimal or no PTV margins. For a 0.5 cm diameter target, even a 0.5° rotation resulted in coverage below 95% at 1.5 cm from the isocenter without a margin, dropping significantly at greater distances. Conversely, larger targets maintained adequate coverage even at 2.5° rotation without a margin. Introduction of a 1.0 mm uniform margin generally ensured over 95% GTV coverage for 1° rotations across all evaluated target sizes and distances from the isocenter. Applying this framework to a clinical case, differential margins derived from our model resulted in a decreased treated volume and reduced brain V12Gy compared to a uniform 1 mm margin plan.

Conclusions

The findings underscore the impact of rotation based on target size and location and the critical importance of individualized PTV margin selection. By leveraging this analysis, clinicians can determine optimal, customized margins based on specific target characteristics and acceptable rotation thresholds, thereby ensuring robust target coverage while minimizing unnecessary irradiation of healthy tissue and enhancing the precision of SIMT treatments.

## Introduction

Volumetric modulated arc therapy (VMAT) has emerged as a revolutionary technique in the field of stereotactic radiosurgery (SRS), offering significant advancements over traditional methods. VMAT's unique capability to treat multiple targets simultaneously using a single plan with one isocenter (single-isocenter multi-target or SIMT) marks a substantial improvement in efficiency and treatment accuracy [1-2]. This advanced technique leverages non-coplanar arcs and the simultaneous variation of multileaf collimator leaf positions, dose rate, and gantry rotation speed to achieve highly conformal dose distributions [3-5].

However, this efficacy is not without trade-offs. When treating multiple targets independently with separate plans, each plan's isocenter can be aligned with a target, thus ensuring minimal dosimetric effects from rotational errors. In contrast, SIMT SRS treatments are less robust against rotational errors. The displacement of a target from the point of rotation increases with its distance from the isocenter, leading to greater dosimetric impacts [6-7].

Rotational errors in patient setup are particularly impactful in SIMT due to the relationship between the diameter of the target and the distance from the isocenter to the target. The effect of these errors on dose coverage necessitates careful consideration of the Planning Target Volume (PTV) margins. Larger PTV margins are often required in single iso VMAT to accommodate the six degrees of freedom (6DoF) setup errors, ensuring that dose coverage for Gross Tumor Volumes (GTVs) meets clinical tolerance values.

In clinical practice, a PTV margin of 1 mm is commonly used to concentrate the radiation on the GTV and minimize dose to surrounding normal tissues. However, as the distance between the target and the isocenter increases, the significance of rotational errors grows, potentially compromising dose coverage. Ono et al. [8] investigated the impact of beam positioning accuracy at off-isocenter positions and found that errors increased with greater distance from the isocenter and with increased couch rotation.

Roper et al. [9] investigated the impact of rotational errors on target coverage in SIMT SRS using VMAT. Analysis of 50 SRS cases revealed that compromised coverage increased with smaller target volumes, larger rotational errors, and greater distance between targets and the isocenter. Palmiero et al. [10] found that clinically observable isocenter misalignment in single-isocenter VMAT SRS resulted in significant loss of target coverage, particularly for smaller tumors (up to 73.1% for GTV and 93.7% for PTV), and potential increases in organ-at-risk doses. Nakano et al. [11] investigated the impact of 6DoF setup errors on the accuracy of single-isocenter SRS for multiple brain metastases. Simulated spherical GTVs of varying diameters and distances from the isocenter were used to assess dose coverage reduction due to setup errors. The results showed that dose coverage reduction and required margins increased with smaller tumor size, greater distance from the isocenter, and larger setup errors, highlighting the importance of accurate patient positioning in this technique. Golkamani et al. [12] used phantom and retrospective imaging data to assess the dosimetric impact of rotational errors on SIMT HyperArc plans. Rotational errors of up to 2° caused significant dose reduction (up to 50% for 5 mm diameter PTVs), particularly for smaller targets further from the isocenter. Chang [13] used statistical models to provide guidance on determining acceptable rotational error thresholds and necessary PTV margin adjustments to maintain desired target coverage probability.

All the above-mentioned work has either used simulations, retrospective analysis, or statistical models in determining the effect of rotations on targets in SIMT radiosurgery. Our method relies on setting up and deriving the mathematical equations to compute the overlap between the target and radiation shapes and determining the effective margins for PTV coverage based on the expected rotations. We propose a 2D and 3D mathematical analysis where both the target and the radiation can be modeled as circles and spheres. By computing the overlap of these shapes, the study aims to show the relationship between margin size, magnitude of rotation, distance of the target from the isocenter, and target size, and to quantify the dosimetric consequences of rotational errors in SIMT SRS treatments. With the help of these models, physicians can choose the appropriate margin size based on the size of the target characteristics, thus mitigating the risks associated with setup errors.

## Materials and methods

**Figure 1:**
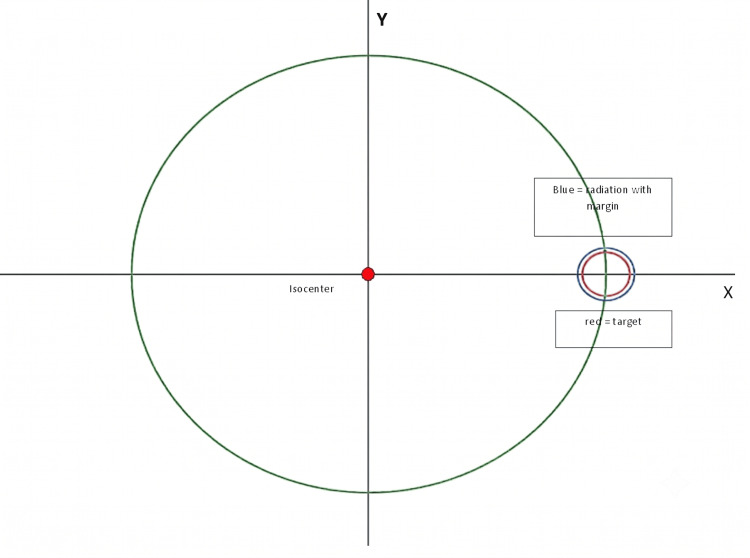
Illustration of the perfect scenario of a target (Gross Tumor Volume = red circle) and radiation (blue circle) overlapping at a distance away from the isocenter. In the perfect scenario, the radiation perfectly encapsulates the target.

The geometry proposed in this study is shown in Figure 1.

The red circle represents the target or the GTV. The larger blue circle represents radiation with a 1 mm margin around the GTV. Assuming the 1 mm margin represents the PTV around the GTV. For simplicity of calculation, the blue circle assumes a 100% coverage of PTV or a PTV V100 = 100% with a conformity index (CI) of 1.0, where CI is defined as

$CI = \frac{\text{Volume covered by the 100\% isodose line}}{\text{Volume of the PTV}}$ (1).

**Figure 2:**
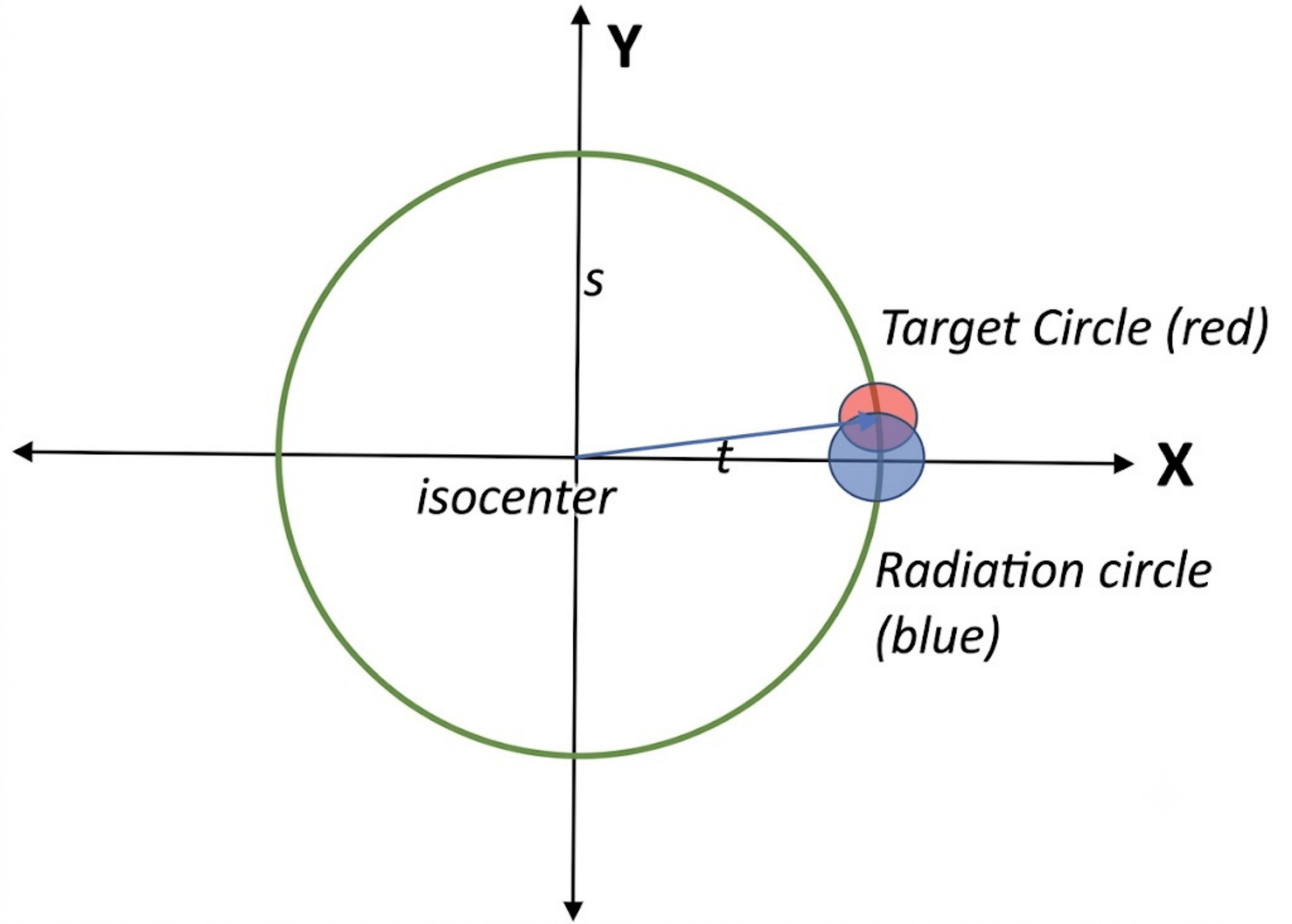
Illustration of the rotation of the patient with respect to the corresponding effect on the radiation treatment. The origin of the axes denotes the isocenter. The big green circle denotes a distance s from the isocenter. The red circle denotes the target (Gross Tumor Volume), which is now rotated by an angle t. The blue circle is the radiation, which is centered on the original location of the target. Because of the target rotation by an angle t, it is not fully covered by the radiation circle.

Figure 2 represents the same scenario as Figure 1 but with a rotation t induced with respect to the target (around the x-axis). Let s be the distance of the target from the isocenter.

If $r_r$ is the radius of the radiation circle, then the equation of the radiation circle is defined as

$C_R = (x-s)^2 + y^2 = r_r^2$ (2).

Let $r_t$ be the radius of the target circle.

The equation of this circle is

$C_T = (x-s\cos t)^2 + (y-s\sin t)^2 = r_t^2$ (3)

$r_r = r_t + m$ (4)

where m is the treatment margin.

Note that if s = 0, so that the target is at the isocenter, then the rotation t does not matter, the two circles will always be coincident.

The area of intersection of the two circles can be calculated as

$A_{intersection} = A_1 + A_2 - A_3$ (5)

where

$A_1 = r_r^2 \cos^{-1}\left(\frac{d^2+r_r^2-r_t^2}{2dr_r}\right)$ (6)

$A_2 = r_t^2 \cos^{-1}\left(\frac{d^2+r_t^2-r_r^2}{2dr_t}\right)$ (7)

$A_3 = \frac{1}{2} \sqrt{(-d+r_r+r_t)(d+r_r-r_t)(d-r_r+r_t)(d+r_r+r_t)}$ (8)

where d is the distance between the centers of the two circles, in our case

$d = \sqrt{(s\cos t - s)^2 + (s\sin t)^2}$ (9).

3D modeling

The above methodology can be extended to three dimensions, where the tumor and the radiation can now be modeled as spheres.

**Figure 3:**
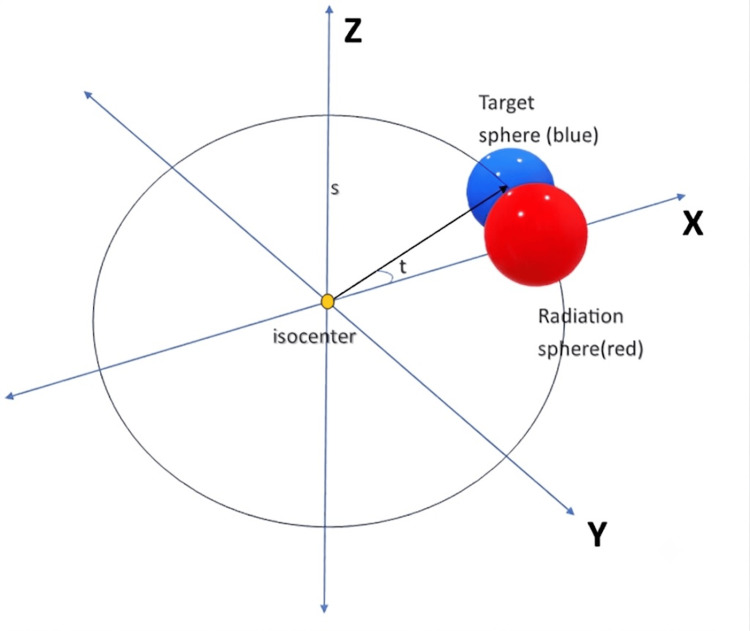
Illustration of the rotation of the patient in 3D. The origin of the axis system is the isocenter. The red sphere denotes the radiation, and the blue sphere shows the met at a distance from s isocenter. There are rotations over multiple dimensions, which cause a significant degradation of the overlap between the Gross Tumor Volume (blue) and the radiation (red).

To calculate the overlap in 3D between the target sphere (GTV) and the radiation sphere, let us assume the target is rotated by t degrees in the X-Y plane and both spheres are centered at (s, 0, 0). This is illustrated in Figure 3 as shown below.

The mathematical approach involves understanding the spatial relationship and the resulting intersection volume between the two spheres.

First, let us define the relevant parameters:

- Let $r_r$ be the radius of the radiation sphere.

- Let $r_t$ be the radius of the target sphere.

- Let s be the distance between the isocenter and the center of the radiation sphere, as well as the center of the target sphere.

The equation of the radiation and target spheres, centered at (s, 0, 0), is given by:

$(x - s)^2 + y^2 + z^2 = r_r^2$ (10)

$(x - s)^2 + y^2 + z^2 = r_t^2$ (11)

- Let t be the angle of rotation of the target sphere with respect to its original position in the X-Y plane.

When the target sphere is rotated by t degrees in the X-Y plane, the coordinates of its center change based on the rotation matrix. The new position of the target sphere can be represented using spherical coordinates and rotation matrices: 

$\left(x-s\cos(t)\right)^2 + \left(y-s\sin(t)\right)^2 + z^2 = r_t^2$ (12).

The distance d between the two sphere centers plays a crucial role in determining the intersection volume and can be computed using the distance formula.

To find the volume of the intersection between the two spheres, we use the formula for the volume of spherical caps. The intersection volume (V) depends on the radii of the spheres and the distance d between their centers. The formula for the intersection volume is approximated as:

$V = \frac{\pi (r_r+r_t-d)^2}{12d} \left(d^2 + 2d(r_r+r_t) - 3(r_r-r_t)^2\right)$ (13).

This intersection volume represents the overlapping region between the target and the radiation sphere, accounting for the rotation and distance from the isocenter. Understanding this overlap helps in computing graphs for varying distances s and angle of rotation t for a target of fixed radius $r_t$.

The value of d leads to specific scenarios for the intersection volume.

For scenarios where $|r_r - r_t| &lt; d &lt; r_r + r_t$, the partial-intersection volume is computed via the derived analytical equations above.

Conversely, if $d + r_t \le r_r$, the target achieves total radiation encapsulation.

The mathematical framework necessitates the distinction of specific geometric conditions: when $d \ge r_r + r_t$, the volume of intersection remains null.

Additionally, the case of d = 0 must be isolated to prevent computational singularities from division by zero.

To model rotation about another axis, say the Y-axis, is simple. Assume the rotation a around the y-axis. The new equation of the target now becomes

$\left(x-s\cos t \cos a\right)^2 + \left(y-s\sin t\right)^2 + \left(z+s\cos t \sin a\right)^2 = r_t^2$ (14).

Once the equation is known, the same formula for the distance between the centers of the two spheres, and subsequently for the volume of overlap V, can be used to generate the overlap of the two new spheres.

Computational implementation

All algorithmic programming, numerical modeling of the 2D/3D geometric intersections, volume computations, and data analysis were performed using Python (Python Software Foundation, Wilmington, DE). Graphical visualizations of GTV coverage as a function of treatment margin across the varied anatomical configurations, as shown in the Results section, were generated utilizing the Matplotlib library (John D. Hunter, Python Software Foundation, Wilmington, DE).

## Results

Target coverage was analyzed with respect to rotational variations for gross tumor diameters of 0.5 cm, 1.0 cm, 2.0 cm, and 3.0 cm. To simplify the initial parametric analysis, simulated rotations were restricted to a single dimension. For each target volume, the off-axis distance from the isocenter ranged from 1.5 cm to 7.5 cm, evaluated in 1.5 cm increments. A horizontal reference line indicating the standard target coverage tolerance of V100% = 95% is included on each plot. The PTV margin was held constant across initial evaluations to isolate the relationship between target diameter and geometric sensitivity to rotation.

**Figure 4:**
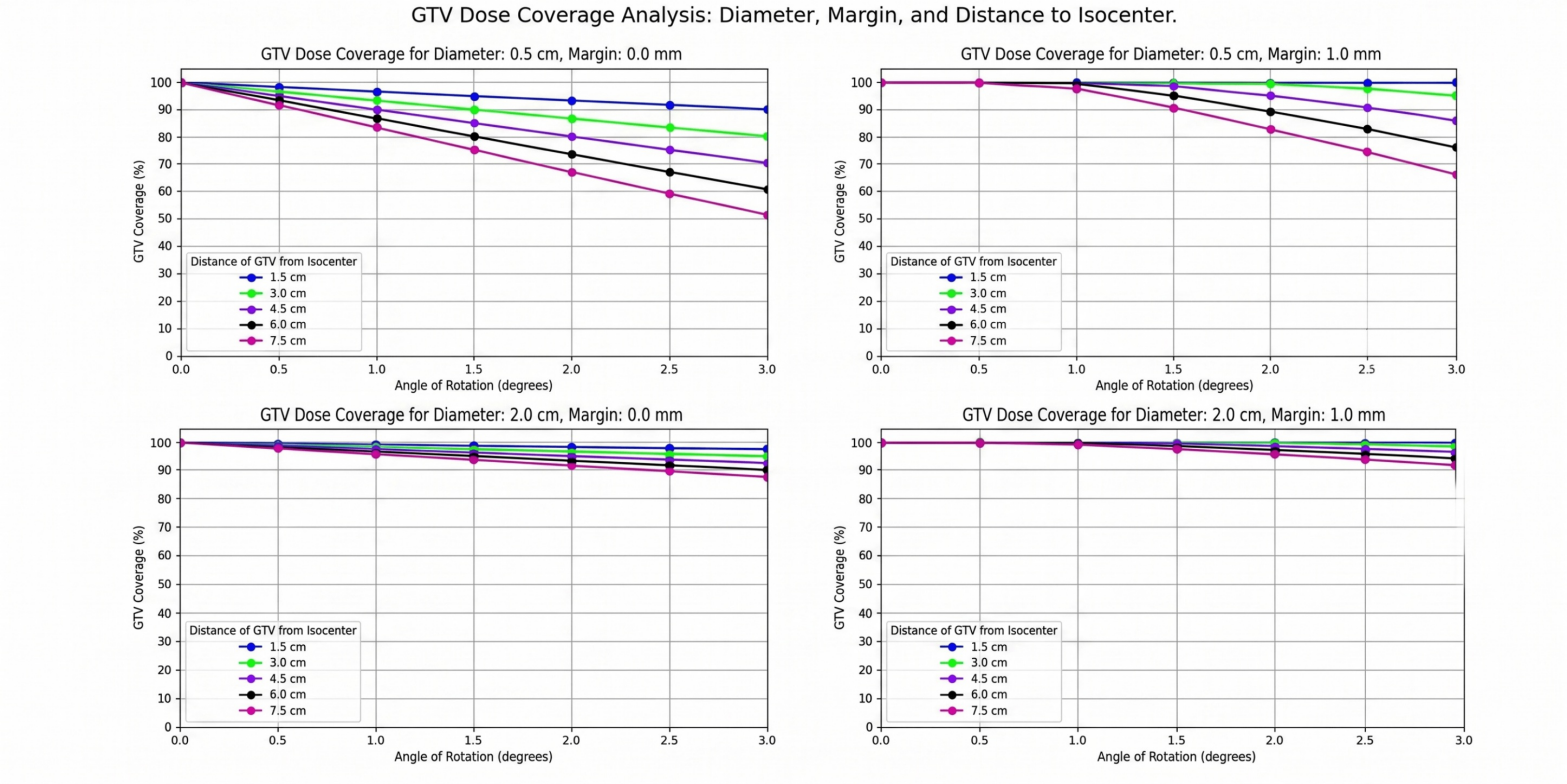
Illustration of proof of concept using 2D geometry. The top row shows the overlap of a radiation circle with the Gross Tumor Volume (GTV) circle for a tumor represented by a circle of diameter 0.5 cm with margins 0 mm and 1 mm, respectively. The bottom row shows the overlap for a GTV represented by a 2.0 cm circle and the same margins.

Figure 4 illustrates the two-dimensional proof-of-concept modeling, wherein both the GTV and the radiation fields are approximated as coplanar circles. The top-left panel displays a 0.5 cm diameter GTV, while the bottom row depicts a 2.0 cm diameter GTV, with each size evaluated at margins of 0 mm and 1 mm. The findings demonstrate that margin addition is uniquely critical for smaller target volumes; for a 0.5 cm GTV positioned at a 3.0 cm radial distance from the isocenter, a minor 1° rotation causes the V100% coverage to fall below the 95% threshold. Coverage degrades precipitously as a function of both increasing off-axis distance and magnitude of rotation. Implementing a uniform 1.0 mm margin effectively preserves adequate coverage (>95%) under a 1° rotation across all clinically relevant off-axis distances. Conversely, the 2.0 cm target exhibits greater intrinsic geometric stability, maintaining a GTV V100% above 90% even at a 2.5° rotation, irrespective of margin inclusion.

While the circular approximation demonstrates the anticipated geometric trends, confirming that smaller, peripheral targets require larger margins, the absolute volumetric coverage values diverge between the two-dimensional and three-dimensional models. Consequently, a three-dimensional assessment utilizing spherical geometry is required to provide clinically applicable insights into margin requirements.

**Figure 5:**
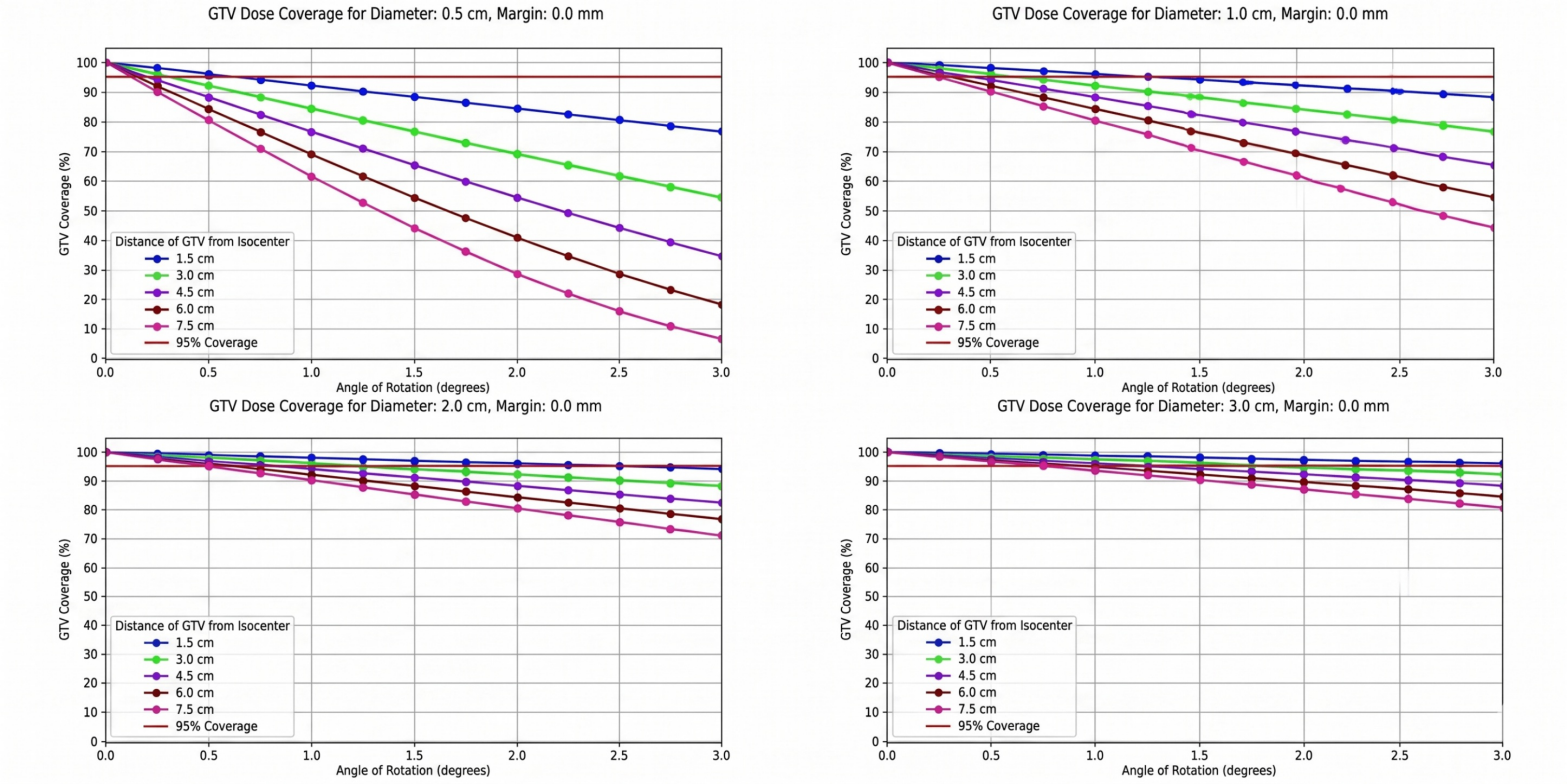
Gross Tumor Volume (GTV) V100% coverage as a function of rotational angle for various target diameters in the absence of a margin (0 mm margin). The target distance from the isocenter varies from 1.5 cm to 7.5 cm in 1.5 cm increments. The horizontal line denotes the V100% = 95% clinical coverage threshold for reference.

Figure 5 shows GTV coverage by radiation versus rotation angle for various target sizes with zero margin. For the smallest target (0.5 cm diameter), even a 0.5° rotation reduces coverage below 95%, even at 1.5 cm from the isocenter. At 7.5 cm, coverage drops from V100 of 80% at 0.5° rotation to under 10% at 3° rotation.

Coverage improves with a larger target size. At 2.0 cm, a 0.5° rotation gives adequate coverage up to 7.5 cm from the isocenter. However, larger rotational displacements cause substantial coverage degradation even for targets as large as 3.0 cm, confirming that larger volumes remain susceptible to significant underdosage as the distance from the isocenter increases.

**Figure 6:**
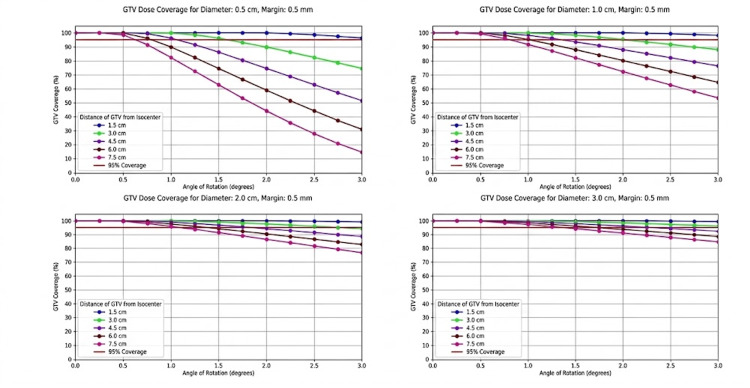
Gross Tumor Volume (GTV) V100% coverage versus rotational angle in the presence of a uniform 0.5 mm Planning Target Volume (PTV) margin. Coverage curves are stratified by target diameter and varied off-axis distances from the isocenter.

Figure 6 illustrates the impact of a 0.5 mm margin on the targets. In comparison to the no-margin scenario shown in Figure 5, the 0.5 mm margin achieves 100% coverage within a 0.5° rotation, including for a 0.5 cm GTV. For smaller targets, this margin provides adequate GTV coverage when the target is located within 3.0 cm from the isocenter and the rotation remains under 1°. For a 2.0 cm diameter target, the 0.5 mm margin maintains sufficient coverage during a 1° rotation even at a 7.5 cm distance from the isocenter. Additionally, with a 2° rotation, the 0.5 mm margin results in 90% GTV coverage for a 3.0 cm diameter target, regardless of its proximity to the isocenter.

**Figure 7:**
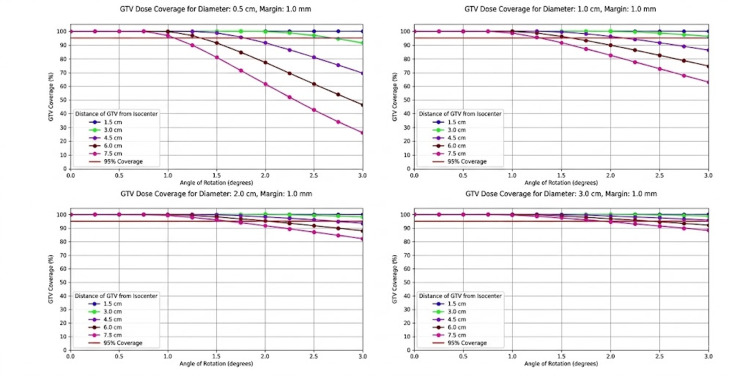
Impact of a uniform 1.0 mm margin on Gross Tumor Volume (GTV) coverage across distinct target diameters and off-axis distances. The horizontal reference line indicates the 95% target coverage threshold.

Figure 7 shows the same targets and rotation, with a 1.0 mm uniform margin. This margin is sufficient to keep the GTV coverage over 95% for a 1° rotation, irrespective of the target size or distance from the isocenter. As the target size increases, it becomes less sensitive to even larger rotations. A 3.0 cm GTV achieves 95% coverage in the presence of a 1.0 mm margin, even at a 2° rotation. For the GTV sizes under consideration, as long as rotations larger than 1° are corrected, a 1.0 mm margin is sufficient for all clinically relevant distances from the isocenter.

**Figure 8:**
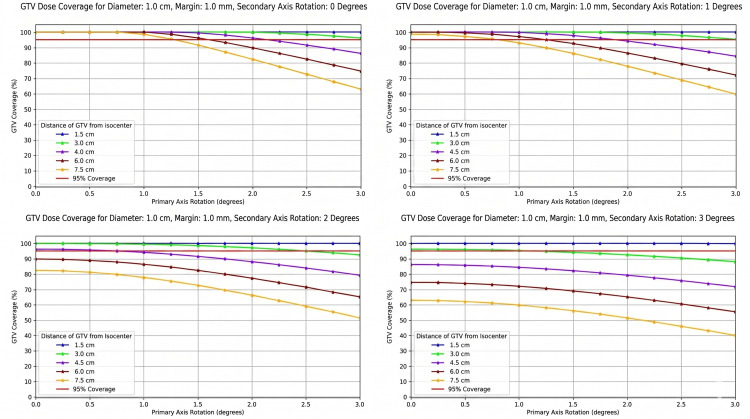
Volumetric Gross Tumor Volume (GTV) coverage under multi-axis rotational setup errors for a 1.0 cm target with a fixed 1.0 mm margin. The baseline plot (top) represents a single-axis rotation (0° secondary rotation), matching the corresponding curve in Figure 7. Subsequent plots simulate the compounding effects of introducing fixed secondary axis rotations of 1°, 2°, and 3°.

Figure 8 illustrates the impact of rotation around two axes for a 1.0 cm GTV with a 1.0 mm margin. In this analysis, secondary axis rotation ranges from 0 to 3°, with the top graph corresponding to the second plot in Figure 7. The results indicate that a 1.0 mm margin is sufficient, provided rotation along a single axis remains within 1°. However, introducing an additional 1° rotation along a different axis reduces coverage at greater distances from the isocenter. Notably, a 2° rotation about one axis yields worse coverage than two separate 1° rotations on different axes. Consequently, as long as each individual rotation is corrected and minimized, a 1.0 mm margin should be adequate. If rotations exceed 2° on any axis, coverage declines rapidly with increasing distance from the isocenter.

**Figure 9:**
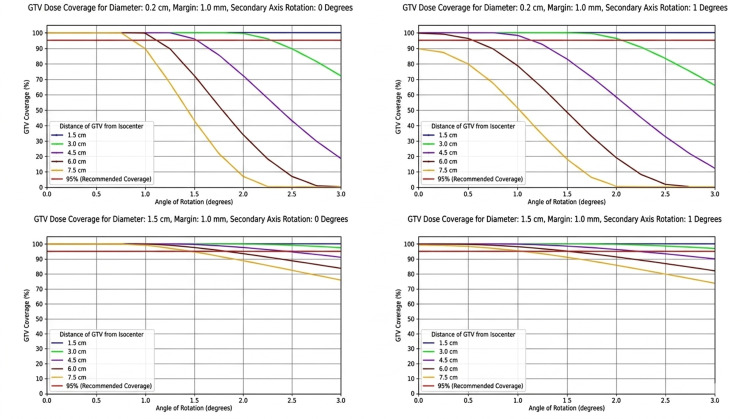
Rotational analysis for alternative target volumes (0.2 cm and 1.5 cm Gross Tumor Volumes (GTVs)) utilizing a fixed 1.0 mm margin. The top row evaluates a 0.2 cm GTV, and the bottom row evaluates a 1.5 cm GTV, modeled under secondary axis rotations of 0° and 1°.

Figure 9 characterizes rotational effects on alternative target volumes. The top panel evaluates a 0.2 cm ultra-small lesion managed with a 1.0 mm margin under secondary axis rotations of 0° and 1°. For lesions of this scale, target coverage is exceptionally sensitive to the off-axis distance. Even with a 1.0 mm margin, GTV coverage falls below acceptable clinical limits at distances exceeding 4.5 cm from the isocenter. A single-axis rotation of 1° reduces the V100% of this 0.2 cm lesion to 90% at a 7.5 cm off-axis distance. In contrast, a 1.5 cm target consistently satisfies the 95% coverage requirement for multi-axis rotations under 1.0° across all evaluated distances.

Clinical application

The above work can be clinically applied to determine the minimum viable margin by inverting the problem. Assume that in a clinical scenario, rotations above a tolerance can be corrected using imaging or surface guidance systems. In such a scenario, for a fixed target diameter, distance from the isocenter, one can compute the minimum margin required such that a fixed GTV coverage (e.g., at least 95% of GTV receiving 100% of the prescribed dose, GTV V100 > 95%) is maintained. By varying the margin, in equations 4 and 13 above, the size of the dose distribution sphere can be adjusted such that there is a maximum overlap for a given acceptable rotation. Tables 1 and 2 show the computed margins for differently sized targets and at a fixed distance from the isocenter. Table 1 assumes rotations above 1° in all three directions can be corrected, and Table 2 shows a worst-case scenario of a minimum correctable rotation of 2°. Both cases compute the margins required for a GTV V100 = 95%. 

**Table 1:** Minimum required Planning Target Volume (PTV) margins (mm) to achieve a Gross Tumor Volume (GTV) V100% > 95% given a maximum allowable rotational setup error of 1°, detailed by target diameter and off-axis distance from the isocenter.

GTV sizes (mm)	Margins required (mm) for distances from isocenter				
	15 mm	30 mm	45 mm	60 mm	75 mm
5	0.2	0.5	0.8	1.1	1.5
10	0.1	0.3	0.6	0.9	1.2
15	0	0.2	0.4	0.7	1.0
20	0	0.1	0.3	0.6	0.8
30	0	0	0.1	0.3	0.6

**Table 2:** Minimum required Planning Target Volume (PTV) margins (mm) to achieve a Gross Tumor Volume (GTV) V100% > 95% given a maximum allowable rotational setup error of 2°, detailed by target diameter and off-axis distance from the isocenter.

GTV sizes (mm)	Margins required (mm) for distances from isocenter				
	15 mm	30 mm	45 mm	60 mm	75 mm
5	0.5	1.1	1.8	2.5	3.2
10	0.3	0.9	1.5	2.2	2.9
15	0.2	0.7	1.3	1.9	2.6
20	0.1	0.6	1.1	1.7	2.3
30	0	0.3	0.8	1.4	1.9

**Figure 10:**
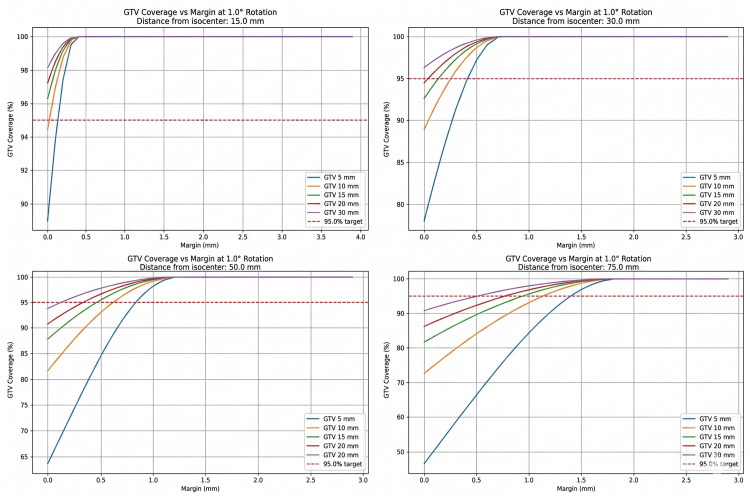
Gross Tumor Volume (GTV) coverage as a function of margin size across various target diameters (5 mm to 30 mm) and distances from the isocenter, assuming an allowable rotational uncertainty threshold of 1°.

The numbers in Table 1 match the results that we have observed so far. Smaller targets further away from the isocenter require larger margins. For a 1° rotation threshold, a 5 mm target 7.5 cm from the isocenter requires a 1.5 mm margin to maintain a GTV coverage of 95%. For the same distance, a 3.0 cm target only requires a 0.6 mm margin. In fact, a 3.0 cm target requires no margin if it is 3.0 cm from the isocenter. In such a scenario, even with 1° rotations, the GTV coverage is maintained. The results from Table 2 show the data for 2° rotation, which is more drastic for smaller targets far away from the isocenter. At 6.0 cm from the isocenter, a 5 mm target requires a 2.5 mm margin on either side, i.e., the scale of the margin matches the target size. The same information is presented in Figure 10, showing the variation of GTV coverage with margin size for different-sized targets at fixed distances from the isocenter. The figures confirm the previous observations that the closer the targets are to the isocenter, the smaller the margin required, and the margin size is inversely proportional to target size.

To validate this variable margin selection strategy, the model was retrospectively applied to ten clinical cases previously treated at our institution. Treatment plans had been generated via the Elements (Brainlab, Munich, Germany) treatment planning system for a Varian Edge linear accelerator (Varian Medical Systems, Palo Alto, CA) equipped with a High-Definition 120-leaf multileaf collimator (HD-120-MLC). To eliminate inter-planner variability, all cases were generated by a single medical physicist (S.N.). The treatment planning system automatically placed the single isocenter at the multi-lesion centroid of the intracranial metastases, and delivery was optimized using dynamic conformal arcs (DCA). Dose calculations were performed on a refined 1 mm isotropic grid, with all targets receiving a uniform prescription dose of 20 Gy in a single fraction. While the original clinical plans utilized a standard, uniform 1.0 mm PTV margin across all lesions, our retrospective evaluation isolated rotational uncertainties under the assumption that rotations above 1° are strictly corrected. Margins were selected according to the calculations in Table 1, applying a baseline floor margin of 0.5 mm to account for residual non-rotational treatment uncertainties (such as translational and machine-specific tolerances).

The cohort of selected patients presented with a metastatic burden ranging from two to eight lesions per case (median: 3; mean: 3.6), with individual lesions situated at off-axis distances between 1.2 cm and 7.1 cm from the isocenter. The maximum diameter of the intracranial metastases ranged from 0.3 cm to 1.3 cm.

Implementing the variable-margin strategy derived from Table 1 yielded a systemic reduction in the total PTV for nine out of the 10 evaluated cases. Under the standard, uniform 1.0 mm margin configuration, the total PTV ranged from 0.546 cc to 2.868 cc. In contrast, the application of variable margins significantly compressed this range from 0.303 cc to 2.661 cc, culminating in an average volumetric target reduction of 19.8%.

This reduction in treated volume directly translated to enhanced sparing of healthy tissue. The total volume of normal brain parenchyma receiving a 12 Gy dose (V12Gy) demonstrated a corresponding 19.0% average reduction when utilizing the variable-margin plans (range: 1.28 cc-8.81 cc) compared to the baseline uniform 1.0 mm margin plans (range: 1.74 cc-9.90 cc).

To illustrate the clinical utility of the proposed algorithm, a representative case study involving a patient with eight distinct intracranial metastases is presented. The individual lesion diameters, their respective off-axis distances from the isocenter, and the algorithmically derived PTV margins are detailed in Table 3. In accordance with our protocol, the calculated variable margins were narrower than or equivalent to the standard clinical margins for six of the eight evaluated lesions.

**Table 3:** Gross Tumor Volume (GTV) statistics for a clinical case where the differential margin scenario was applied. The case had eight metastases treated in a single isocenter, with a prescription dose of 20 Gy in a single fraction.

GTV number	GTV diameter (mm)	GTV distance from isocenter (cm)	Margin used (mm)
01	6	5.0	1.1
02	6	4.7	1.0
03	5	5.0	1.0
04	4	3.0	0.5
05	11	3.5	0.5
06	9	5.5	0.8
07	13	6.4	1.0
08	5	6.3	1.1

A dosimetric comparison between the treatment plans demonstrated that the total PTV decreased from 2.816 cc to 2.661 cc when transitioning from uniform to differential margins. Crucially, this volumetric reduction directly reduced normal tissue toxicity, with the normal brain volume receiving 12 Gy (V12Gy) decreasing from 9.9 cc to 7.4 cc in the differential-margin plan.

As these metrics indicate, the differential-margin technique offers a robust mechanism for minimizing healthy brain parenchymal dose and maximizing tissue preservation. Simultaneously, it ensures that target coverage remains resilient against rotational setup uncertainties, provided these deviations are actively corrected within the established angular tolerances.

## Discussion

We mathematically calculated and evaluated the effect of rotational errors on targets away from the isocenter as a function of distance from the isocenter, target size, and size of applied margin. The result shows that the target size is the biggest predictor of loss of coverage, with distance from the isocenter being equally important. A large target is more robust to loss of coverage due to rotation, even at distances further from the isocenter. This is especially the case if a typical 1.0 mm margin is applied to the GTV to create a PTV. For smaller targets, however, the coverage loss becomes drastic as the distance from the isocenter increases, even in the presence of margins.

To our knowledge, this is the first study that has performed a closed-form analytical derivation of the effect of patient rotations on single iso-multi target radiosurgery. Prior work on this topic can be divided into two main categories: studies based on simulated patient data [7,9,10,12] and geometric [11] or statistical [13] modeling of patient rotations.

Sagawa et al. [7] analyzed 29 real patients with one to eight brain metastases. They computationally introduced rotational errors by shifting coordinates to re-calculate the plans. Roper et al. [9] evaluated 50 actual clinical patient cases (each with two targets). They artificially applied 0.5°, 1.0°, and 2.0° rotations across all axes inside the planning system to perform a multivariate regression. Palmiero et al [10] utilized real patient VMAT SRS datasets to simulate 6DoF isocenter misalignments, tracking the exact drop in GTV/PTV coverage and unintended dose to organs at risk (OARs). Golmakani et al. [12] combined a retrospective patient imaging analysis with physical phantom measurements to see how real HyperArc plans hold up against simulated shifts.

Nakano et al. [11] used mathematically simulated, perfect spherical GTVs (ranging from 1.0 to 3.0 cm in diameter) and rotational vectors to analytically solve for the maximum distance a target can be from the isocenter before geometric coverage drops below a 95% tolerance threshold. While our study closely follows the analysis performed by Nakano’s group [11], there are some significant differences. Our study created a closed-form solution to compute volume overlap and coverage loss, as compared to a point-by-point boundary check and iterative volume reduction computation used by Nakano et al. [11]. Chang [13] developed a robust statistical model to analytically predict how rotational uncertainties translate into positional errors across multiple off-isocenter targets. This mathematical framework provides the theoretical foundation necessary to translate discrete geometric setup variations into probabilistic dosimetric outcomes during the planning phase. This was made possible by the assumption that the 3D rotational setup errors follow independent normal distributions around the isocenter.

Finally, Ono et al. [8] used a physical linear accelerator in developer mode with a specialized phantom to automatically measure the actual mechanical and beam-positioning alignment errors when moving away from the central isocenter. Thus, this study differed from the abovementioned classification in the absence of the use of patient data or mathematical derivations.

Roper et al. [9] and Nakano et al. [11] have both indicated that the impact of rotations is minimal if such rotations are maintained below 0.5°. The data presented in Figures 5-7 support this assertion, provided there is a margin of at least 0.5 mm. SRS of brain metastasis is typically performed with a 1.0 mm margin added to the GTV, thus creating the PTV. In the context of SIMT radiosurgery, the margin is of increased significance, as targets located further from the isocenter are more vulnerable to coverage loss due to rotational deviations. Studies by Sagawa et al. [7], Roper et al. [9], and Nakano et al. [11] all showed the need for target-specific margins. Our own analysis and data presented in Tables 1-3 again support this finding.

For smaller targets further away from the isocenter, a 1.0 mm margin may not be sufficient, whereas for larger targets that are closer to the isocenter, a 1.0 mm margin may be an overkill. Using too large a margin is not ideal since this leads to irradiation of more normal brain tissue, potentially leading to radiation necrosis. The mathematical models developed in this paper allow the user to customize the margin based on target size and distance from the isocenter. For small distal metastases, adding target-specific margins causes a disproportionate increase in the normal brain tissue volume irradiated. If a peripheral target requires a margin expansion so large that it compromises normal tissue constraints, the correct clinical decision would be to abandon the single-isocenter technique for that lesion and generate a separate, dedicated isocenter plan. Our results establish a practical guide for computing margin expansions tailored to target size and distance from the isocenter, facilitating informed decision-making regarding single-isocenter versus multi-isocenter execution.

When treating multiple brain metastases with a single isocenter, the ability to monitor and correct for patient movement during treatment with real-time monitoring becomes crucial. This becomes particularly relevant when treating small targets that are distant from the isocenter. The margin size and the distance of targets, particularly smaller ones, from the isocenter, will also depend on the real-time monitoring capabilities of the facility where the treatment is being conducted. ExacTrac Dynamic (Brainlab, Munich, Germany) or AlignRT (Vision RT, London, UK) are two examples of systems that are capable of real-time monitoring and corrections. Our center uses ExacTrac Dynamic, which uses both surface guidance and real-time X-rays to monitor patient movement. If the patient's setup deviates by more than 0.5 mm or 0.5°, the treatment will be interrupted, and necessary corrections will be applied. This enables us to keep our margin sizes small even when the targets are far away from the isocenter. Since rotations of approximately 0.5° can be corrected, PTV margins can be determined based on prior knowledge of target size and distance from the isocenter during planning.

The primary limitation of this study is that it assumes that the targets are spherically shaped. This assumption is necessary to generate equations and perform mathematical modeling of the effect of rotations on coverage. The spherical assumption would be valid for small targets, since the smaller the tumor gets, the more spherical it becomes. As the tumor size increases, they become more irregular, and the approximation fails. However, the study can still be used to create a worst-case understanding of the effects of rotations and estimate the margins necessary for these larger tumors. Another limitation is that the study was performed using one- and two-axis rotations. The equation for rotations can be expanded to all three axes; however, the visualization of results will be complex since now there are three different variables to contend with. The single-axis rotation is the best-case scenario, and if the margin is insufficient or the distance from the isocenter is too large for this case, it will certainly fail for the multiple-axis case. Such information can thus be used for prudent clinical judgment. Finally, this is a study that only considers rotational errors, and the translational component of setup errors was not considered. However, in the absence of rotation, the effect of translational errors is uniform across all targets, irrespective of the distance from the isocenter. Furthermore, as translational errors (~1 mm) are an order of magnitude smaller than the typical target distance from the isocenter (~5 cm), any residual uncorrected translational error has a negligible impact on rotational coverage loss for peripheral targets. Using a margin to account for these setup errors and then correcting translational shifts as they are observed during treatment should ensure that the coverage for the GTV is maintained.

## Conclusions

In conclusion, we derived mathematical models to estimate target coverage for various rotations based on spherical geometry. The models estimate the coverage as a function of target size, distance from the isocenter, the margin used, and the degree of rotation. Our models indicate that rotational errors significantly influence target coverage. The resulting graphs serve as a practical clinical tool for rapidly assessing coverage probabilities during the treatment planning phase. By utilizing prior knowledge of each target's size and distance from the isocenter, we can determine custom margins tailored to the tolerable and correctable error for each target. Importantly, these models serve as a predictive tool to guide the choice between single and multiple isocenters during the planning phase. Rather than uniformly expanding margins for distant lesions, clinicians can identify the exact spatial limits when splitting the plan into separate isocenters, which becomes the superior dosimetric choice. These estimates enable precise administration of SIMT radiosurgery, ensuring the preservation of healthy brain tissue.
